# Krüppel-like Factor 5 Promotes Sonic Hedgehog Signaling and Neoplasia in Barrett's Esophagus and Esophageal Adenocarcinoma

**DOI:** 10.1016/j.tranon.2019.07.006

**Published:** 2019-08-08

**Authors:** Christopher K. Ng, Ke Ma, Yulan Cheng, Tomoharu Miyashita, John W. Harmon, Stephen J. Meltzer

**Affiliations:** * Division of Gastroenterology and Hepatology, Johns Hopkins University School of Medicine, Baltimore, MD, USA; † Department of Gastroenterological Surgery, Kanazawa University Hospital, Kanazawa, Japan; ‡ Department of Surgery, Johns Hopkins University School of Medicine, Baltimore, MD, USA

## Abstract

Krüppel-like Factor 5 (KLF5) is a zinc-finger transcription factor associated with cell cycle progression and cell survival. KLF5 plays a key role in mammalian intestinal epithelium development and maintenance, expressed at high levels in basal proliferating cells and low levels in terminally differentiated cells. Considering Barrett's esophagus (BE) and esophageal adenocarcinoma's (EAC) histopathological similarities to intestinal epithelium, we sought to determine KLF5’s role in BE and EAC, as well as KLF5’s possible connection to the sonic hedgehog (SHH) pathway which is highly active in BE and EAC development. Low levels of KLF5 mRNA were found in BE cell lines and tissue– similar to what has been reported in differentiated intestinal epithelium. In contrast, higher KLF5 levels were observed in EAC cells and tissues. KLF5 knockdown in EAC cells caused significant decreases in cell migration, proliferation, and EAC-associated gene expression. Moreover, KLF5 knockdown led to decreased SHH signaling. These results suggest that KLF5 is connected to the SHH pathway in BE and EAC and may represent a potential drug target in EAC; further studies are now indicated to verify these findings and elucidate underlying mechanisms involved.

## Introduction

Esophageal cancer (EC) is the sixth leading cause of cancer death worldwide, and the eighth most common cancer, with roughly 450,000 new cases annually [1]. Outcomes of esophageal cancer are generally poor, with five-year survival rates of approximately 17% [1]. This poor outcome is thought to be due to late diagnosis and complications caused by comorbidity associated with EC [2]. EC is classified into two major categories: esophageal squamous cell carcinoma (ESSC) and esophageal adenocarcinoma (EAC). ESSC arises from the normal squamous esophageal epithelial cells, whereas EAC is thought to arise from Barrett's esophagus (BE) [3]. BE is a condition in which metaplastic mucus-secreting columnar intestinal-type epithelium replaces the normal stratified squamous epithelium of the distal esophagus; in the United States, BE is defined histologically by the presence of intestinal metaplasia, specifically goblet cells [4], [5]. BE and EAC are phenotypically similar to intestinal epithelial tissue and colon cancer, respectively, expressing high levels of intestine-specific transcription factors such as CDX1, CDX2, MUC2, MUC5ac, and VIL [4], [5], [6], [7], [8], [9], [10], [11]. While only 5% of EAC diagnoses are preceded by a BE diagnosis, patients diagnosed with BE are at an 11.3-fold greater risk of developing EAC than those without BE, and underreporting of BE in the population is likely [12], [13].

**Figure 1:**
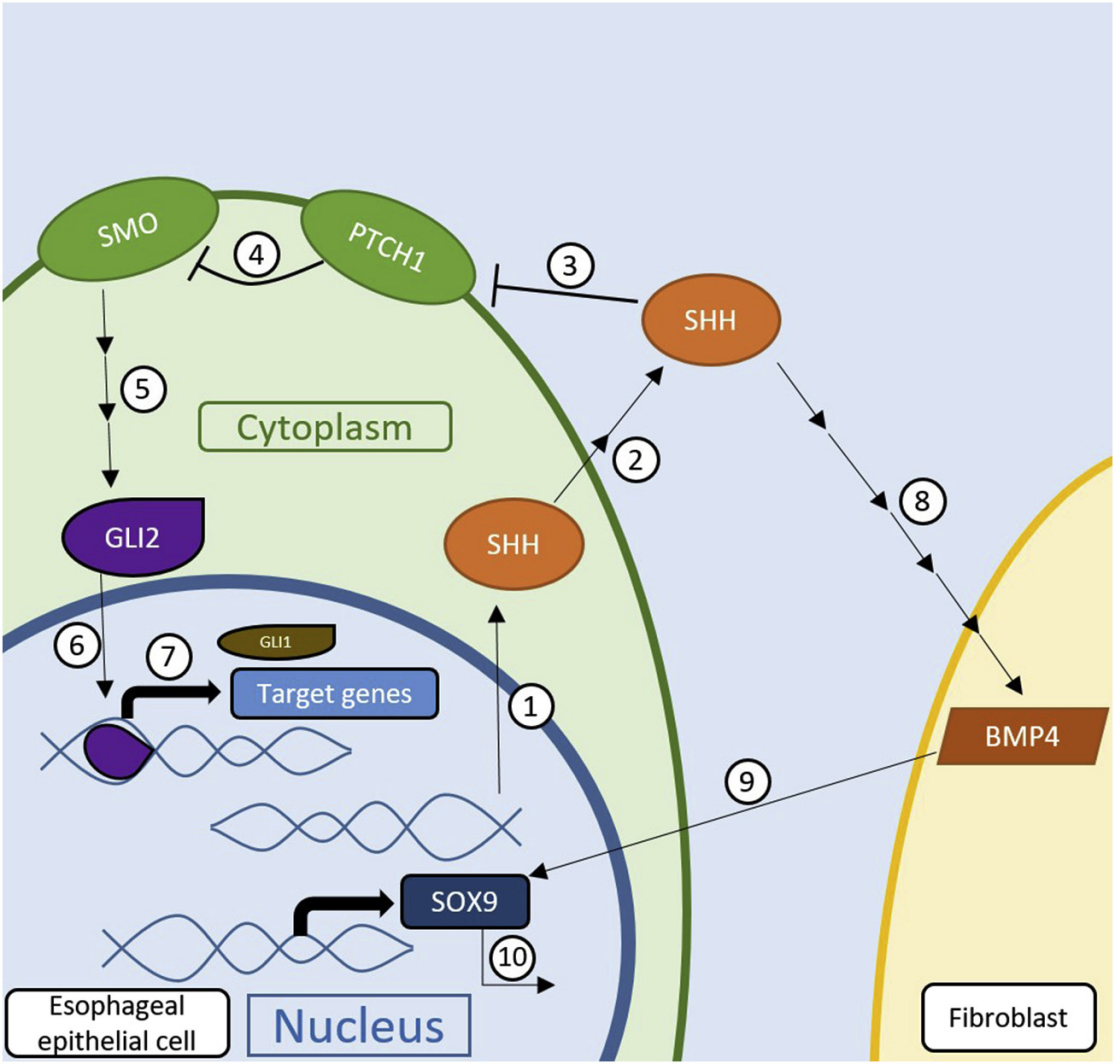
**SHH pathway activity leading to phenotypical changes in esophageal epithelial cells.** 1. In the esophageal epithelium, SHH is expressed in response to damage caused by acid reflux. 2. SHH is modified and exported out of the epithelium. 3. SHH binds to and inhibits PTCH1 4. Due to inhibition by SHH, PTCH1 inhibition of SMO is released and SMO is activated. 5. Activated SMO initiates a signaling cascade that leads to the activation of GLI2. 6. GLI2 translocate to the nucleus. 7. GLI2 activity in the nucleus ultimately leads to the transcription of SHH pathway targets, as well as intestinal phenotypes such as CDX1 and CDX2. 8. SHH begin the previously mentioned steps (3–7) in a neighboring fibroblast where SHH induces BMP4 translation. 9. BMP4 is exported out of the fibroblast and feeds back to the esophageal epithelial cell. 10. In response to fibroblast-created BMP4, SOX9 is expressed in the esophageal epithelial cell where it is sufficient to drive columnar differentiation of squamous epithelium and expression of intestinal markers.

The sonic hedgehog (SHH) signaling pathway is involved in embryonic development, cell proliferation, tissue polarity, and carcinogenesis [14]. Canonically, SHH protein is exported out of the cell, where it will bind and inhibit PTCH1 on neighboring cells or the cell of origin; the inhibition of PTCH1 releases the inhibition of SMO, and activated SMO initiates a signaling cascade that ultimately activates GLI2 proteins in the cytoplasm; finally, activated GLI2 translocates to the nucleus, allowing SHH target genes to be expressed (Figure 1) [15], [16]. SHH pathway activity is known to be upregulated in BE and EAC [17], [18]. Moreover, SHH expression is induced in esophageal epithelial tissue exposed to acid and bile [18]. In esophageal epithelial tissue, SHH released into the extracellular matrix is targeted towards stromal fibroblasts, where it induces secretion of BMP4, which in turn feeds back to the epithelium, causing SOX9 upregulation; SOX9 is sufficient to drive columnar differentiation of squamous epithelium and expression of intestinal markers (Figure 1) [18], [19], [20]. Many studies have investigated the SHH pathway as a cancer drug target using cyclopamine, vismodegib, itraconazole, and other SHH pathway modulators [14], [21], [22]. Clinical trials have revealed varying success with SHH pathway-targeting drugs in different cancer types [14].

**Figure 2:**
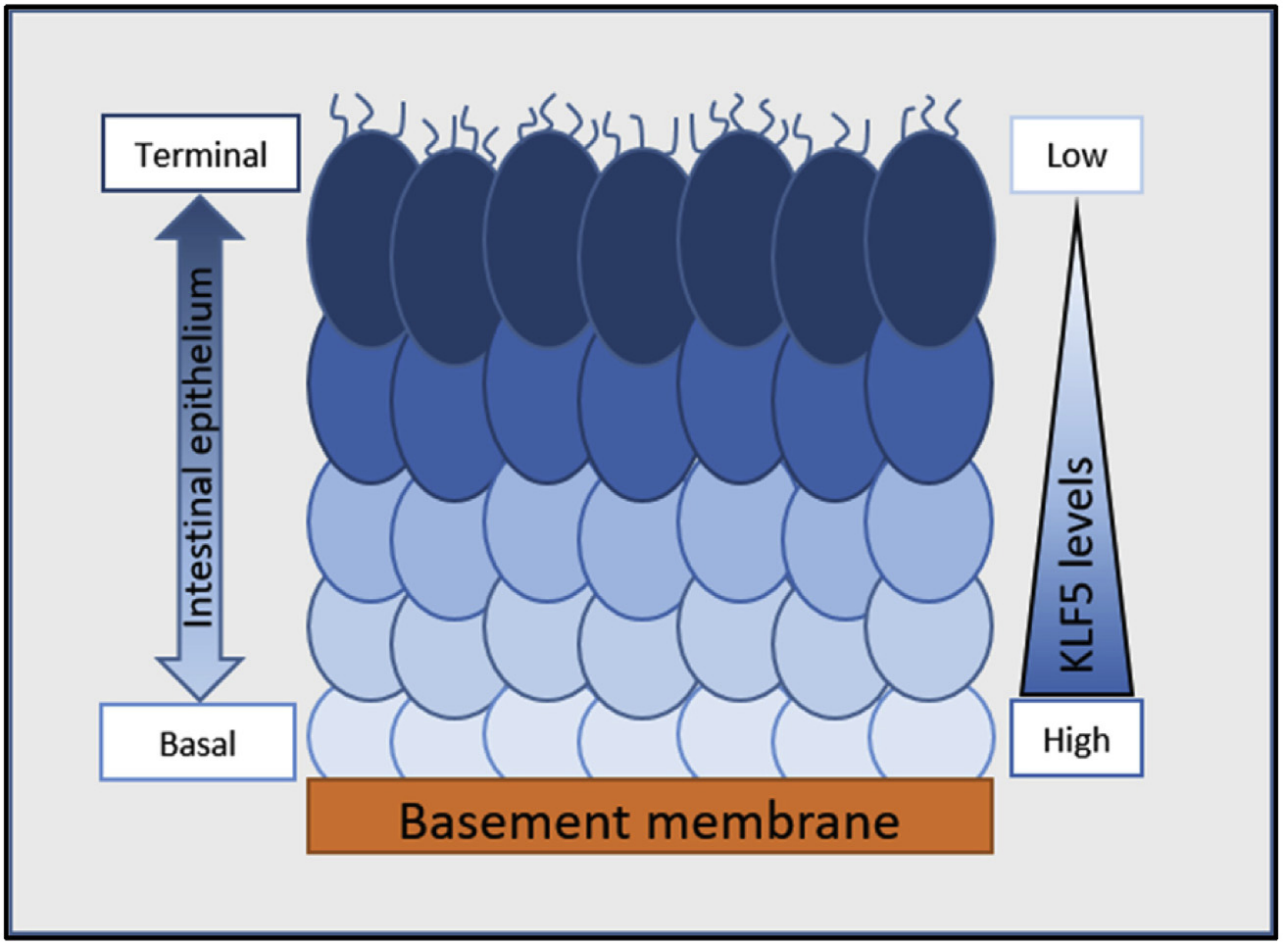
**KLF5 levels in the intestinal epithelium.** In intestinal epithelium, KLF5 is highly active in the undifferentiated basal cells; KLF5 is less active in the terminally differentiated cells.

Krüppel-Like Factor 5 (KLF5) is a zinc finger-containing transcription factor that is highly active in less-differentiated basal intestinal epithelial cells in adult mammals; in contrast, terminally differentiated intestinal epithelial cells express low levels of KLF5 (Fig. 2) [23], [24], [25]. In germline KLF5-deficient mice, intestinal crypts are severely distorted, with reduced numbers of goblet cells [24]. In another murine study, KLF5^Δ/Δ^ intestines failed to form villi, despite expressing factors known to mediate epithelial-mesenchymal signaling essential for villus formation, including SHH, PTCH1, GLI2, and BMP4 [26]. Based on these findings, KLF5 is widely believed to play a key role in intestinal epithelial identity and maintenance [27].

KLF5 activity stimulates cell cycle progression by upregulating cyclins D1 and B2 and downregulating p15 and p27 [28], [29], [30]. Moreover, KLF5 activity suppresses apoptosis in both a p53-dependent and -independent manner [31], [32]. Because of these potentially oncogenic properties, KLF5 activity has been widely investigated and found to be dysregulated in cancers of the pancreas, stomach, breast, prostate and esophageal squamous cell carcinoma; in particular, upregulated KLF5 activity has been extensively studied in colorectal cancer [33], [34], [35], [36], [37], [38]. A comparative genomic study was performed on EAC and ESSC, and the region that harbors KLF5 was found to be amplified in 17% of EAC samples and 0% of ESSC samples; conversely, the same region was deleted in 0% of EAC samples and 20% of ESSC samples [39]. Consistent with this genomic study, KLF5 has been shown to act as a tumor suppressor gene in ESSC; however, to our knowledge, a focused study on KLF5's role in EAC has not yet been performed [33].

In view of EAC's known pathophysiological and histological kinship to both normal and neoplastic intestine, we investigated the involvement of the known enterogenic gene KLF5 in EAC [3]. Moreover, because of the known involvement of the SHH pathway in EAC, we also sought to determine whether KLF5 activity promoted EAC by potentiating SHH signaling.

## Methods and Materials

### Cell Culture

Primary normal non-immortalized esophageal epithelial cells (HEEpiC) were purchased from ScienCell Research Laboratories (Carlsbad, USA). The EAC cell lines SKGT4 and OE33 were purchased from ATCC (Manassas, VA). The telomerase-immortalized primary BE cell lines GrhTRT and QhTRT were generous gifts from Dr. Peter Rabinovitch, Fred Hutchinson Cancer Center, Seattle, WA. HEEpiC was grown in low-serum medium supplemented with growth factors (ScienCell Research, Carlsbad, USA); all other cell lines were grown in media supplemented with 10% fetal bovine serum (Invitrogen, San Diego, USA).

### Clinical Tissues

Human biopsy tissues were obtained during endoscopy performed for clinical diagnostic indications and stored in liquid nitrogen prior to RNA extraction. All patients provided written informed consent under protocols approved by institutional review boards at the Johns Hopkins University School of Medicine, the University of Maryland School of Medicine, or the Baltimore Veterans Affairs Medical Center. All tissues were histopathologically confirmed as normal esophagus (NE), Barrett's esophagus (BE), or esophageal adenocarcinoma (EAC). Thirty-three matched pairs of NE-BE and twenty-eight of NE-EAC were available for quantitative real-time PCR.

### Rat Tissues

The Institutional Animal Care and Use Committee of the Graduate School of Medical Science, Kanazawa University approved these animal procedures.

Twelve male Wistar rats, each weighing ≈250 g, were used in this study. The animals were housed 3 per cage and maintained at a constant room temperature of 22 ± 3 °C and 55 ± 5% humidity with a 12-hour light–dark cycle. They were randomly divided equally into two groups: mock-surgery group and surgery group. They were all fed standard solid chow (Charles River, Japan).

After a 24-hour fast, an upper abdominal incision was made under diethyl-ether inhalation anesthesia. Briefly, the esophagus was mobilized, preserving the vagus nerves and vasculature of the neck. A loop of jejunum was then identified 4 cm from the ligament of Treitz. The gastroesophageal junction was divided, and an end-to-side anastomosis was performed between the distal esophagus and jejunum, as previously reported [40]. Rats in the mock-surgery group only underwent upper abdominal incisions, which were promptly sutured.

The animals were killed by diethyl-ether inhalation 9 months after surgery, after which the abdomen was opened. A ligature was placed around the afferent and efferent jejunal loops near the esophago-jejunal anastomosis. The esophagus was ligated at the level of the thyroid cartilage through a thoracotomy. The esophagus and the anastomosed jejunum were then removed.

After the specimen was opened longitudinally, two 1 mm wide longitudinal slices of the esophageal mucosa were immediately frozen and stored at −80 °C for RNA extraction and subsequent qRT-PCR. The remaining samples were fixed in 10% formalin for 24 h and then cut at 2 mm intervals along the longitudinal section. The samples were embedded in paraffin for hematoxylin and eosin staining and immunohistochemistry.

### RNA Extraction and Quantitative Real-Time PCR

Total RNA was extracted from cells and tissue with TRIzol (Invitrogen, Frederick, USA) according to the manufacturer's protocol. 500 ng of total RNA was used for reverse transcription with the High-Capacity cDNA Reverse Transcription Kit (Applied Biosystems, Waltham, USA). Quantitative real-time PCR was performed using iQ SYBR Green Supermix (BioRad, Hercules, CA), and measured with an ABI 7900 Sequence Detector. Sequences of the primers used are shown in Supplementary Table 1. The fold change in expression of target mRNA relative to GAPDH mRNA was calculated based on the threshold cycle (C_T_) for amplification as 2^Δ(ΔCT)^, where ΔC_T_ = C_T, target_ - C_T, GAPDH_. Equal variance Student's t-tests were performed on the linear values ΔC_T_ for statistical purposes. If a standard was available, relative expression levels were obtained by dividing the target gene concentration by GAPDH concentration; then, matched-pair Student's t-tests were performed on BE *vs.* NE or EAC *vs.* NE relative expression levels.

### siRNA Transfection

SKGT4 cells were cultured in 6-well plates with an initial cell count of 2.5x10^5^ per well. Upon reaching 60–80% confluence, the cells were transfected with Lipofectamine RNAiMAX transfection reagent (Thermo Fisher Scientific, Waltham, MA) to deliver KLF5 specific siRNA according to the manufacturer's protocol. The sequence of KLF5 siRNA was 5′-GAUUACCCUGGUUGCACA-3′ (Dharmacon, Lafayette, CO). Cells were also transfected with a negative nonspecific control siRNA (si-NC) (Dharmacon, Lafayette, CO). The appropriate volume of DEPC-treated water was used instead of siRNA for vector-only groups.

### Cell Proliferation Assays

Untreated cells and transfected SKGT4 cells were seeded into 96-well plates with an initial cell count of 1500 cells per well. Cell proliferation was measured 0 hours, 24 hours, 72 hours, and 120 hours after siRNA transfection, using Cell Proliferation Reagent WST-1 (Roche, Basel, Switzerland). At the time of assessment, 10 μl of the reagent added to each well and incubated at 37 °C for 2 hours, and optical density was measured at 660 nm (background) and 440 nm (signal) using a SpectraMax Plus 384 Microplate Reader (Molecular Devices, San Jose, USA). Statistical analyses were performed using one-way ANOVA with Tukey–Kramer post hoc test.

### Colony Formation

One thousand untreated cells and transfected SKGT4 cells each were seeded onto 6-well plates and cultured for 10 days, after which each well was washed twice with PBS. Cells were sequentially stained with Diff-Quik Fixative, Diff-Quik Solution I, and Diff-Quik Solution II (Dade Behring Inc., Newark, DE) for 10 minutes each at room temperature. Colony counting software (OpenCFU) was used to analyze each well.

### *In Vitro* Scratch Assays

*In vitro* scratch assays were used to assess cell migration as described [41]. Untreated cells and transfected cells were transferred to a 6-well plate and grown to 90% confluence. Then, a linear wound was created using a 200 μl pipet tip to scratch the cell monolayer. Images were captured at 0, 24, and 48 hours after wound formation. Wound widths were photographed and measured using Image J software for calculation of healing rate.

### Immunocytochemistry

SKGT4 with and without siRNA transfection were seeded into 4-well Nunc Lab-Tek II Chamber Slides (Thermo Fisher Scientific, Waltham, MA). After reaching 90% confluence, the cells were fixed in acetone at −20 °C for 7 minutes, washed three times with 1xPBS, and then incubated with primary antibodies for GLI1. Subsequently, the cells were incubated with AlexaFluor goat anti-rabbit secondary antibodies. The cells were counterstained with Hoechst 33342 (Thermo-Fisher Scientific), embedded with paraffin, and observed with a Zeiss Axio Observer Inverted Microscope (Zeiss, Oberkochen, Germany) for fluorescent imaging.

## Results

### Gene Expression Levels in BE and EAC Cell Lines

**Figure 3:**
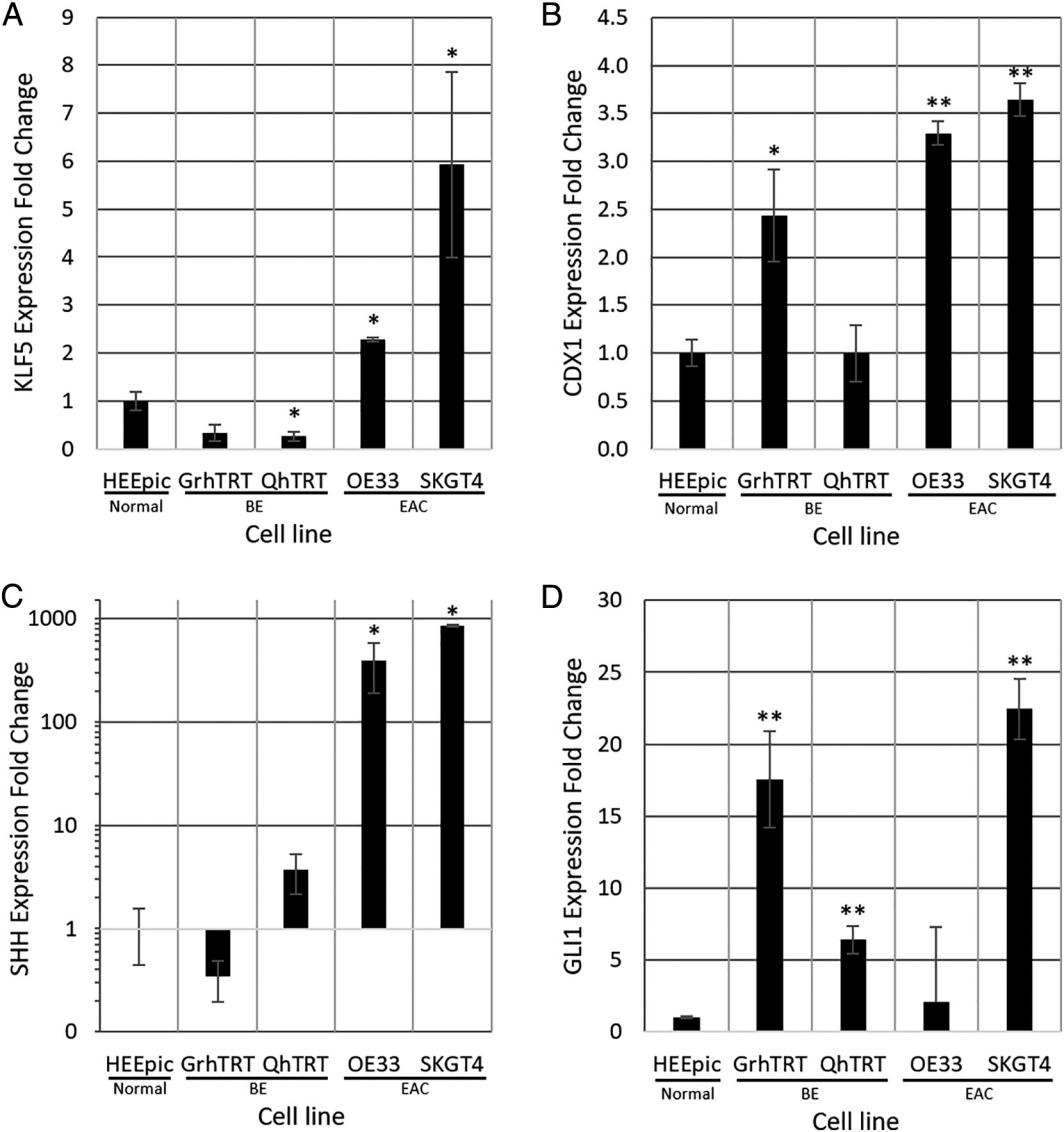
**Baseline KLF5 expression in BE and EAC cell lines.** qRT-PCR was performed on 1 primary normal non-immortalized esophageal epithelial cell line (HEEpiC), two telomerase-immortalized primary BE cell lines (GrhTRT and GhTRT), and two EAC cell lines (OE33 and SKGT4). Expression level was normalized to HEEpiC. Error bars represent standard deviations. A) KLF5 RNA expression was decreased in BE cell lines, GrhTRT and QhTRT, and was elevated in EAC cell lines, OE33 and SKGT4. B) CDX1 RNA expression was increased in GrhTRT, OE33, and SKGT4. C) SHH RNA expression was not significantly changed in both BE cell lines, but was significantly increased in both EAC cells lines. D) GLI1 RNA expression was elevated in all BE and EAC cell lines. n = 4; ∗ *P* < .05; ∗∗ *P* < .005.

We first sought to determine whether KLF5 was differentially expressed in BE and EAC cell lines compared to immortalized normal esophageal squamous epithelial cells (HEEpiC). KLF5 mRNA levels were significantly higher in both EAC cell lines (OE33 and SKGT4) *vs.* HEEpiC (Figure 1*A*). Interestingly, we found significantly lower KLF5 mRNA levels in both BE cell lines (GhTRT and QhTRT) *vs.* HEEpic (Figure 3*A*).

CDX1 is an intestine-specific transcription factor that is also expressed in BE and EAC [6]. As expected, CDX1 mRNA levels were significantly elevated in the GhTRT BE cells, OE33 EAC cells, and SKGT4 EAC cells *vs.* HEEpic normal esophageal cells (Figure 3*B*).

SHH pathway genes are known to be highly active in BE and EAC [17], [18]. To confirm this and to select a cell line for functional experiments, we measured expression levels of SHH and GLI1 in BE and EAC cells. Significantly higher levels of both SHH and GLI1 mRNA were seen in GhTRT and QhTRT BE cell lines as well as in SKGT4 EAC cells *vs.* HEEpic normal cells. Higher levels of SHH and GLI1 were observed in OE33 but were not significantly different from levels in HEEpiC (Figure 3, *C* and *D*).

### KLF5 and the Intestinal Phenotype in Murine BE Tissues

**Figure 4:**
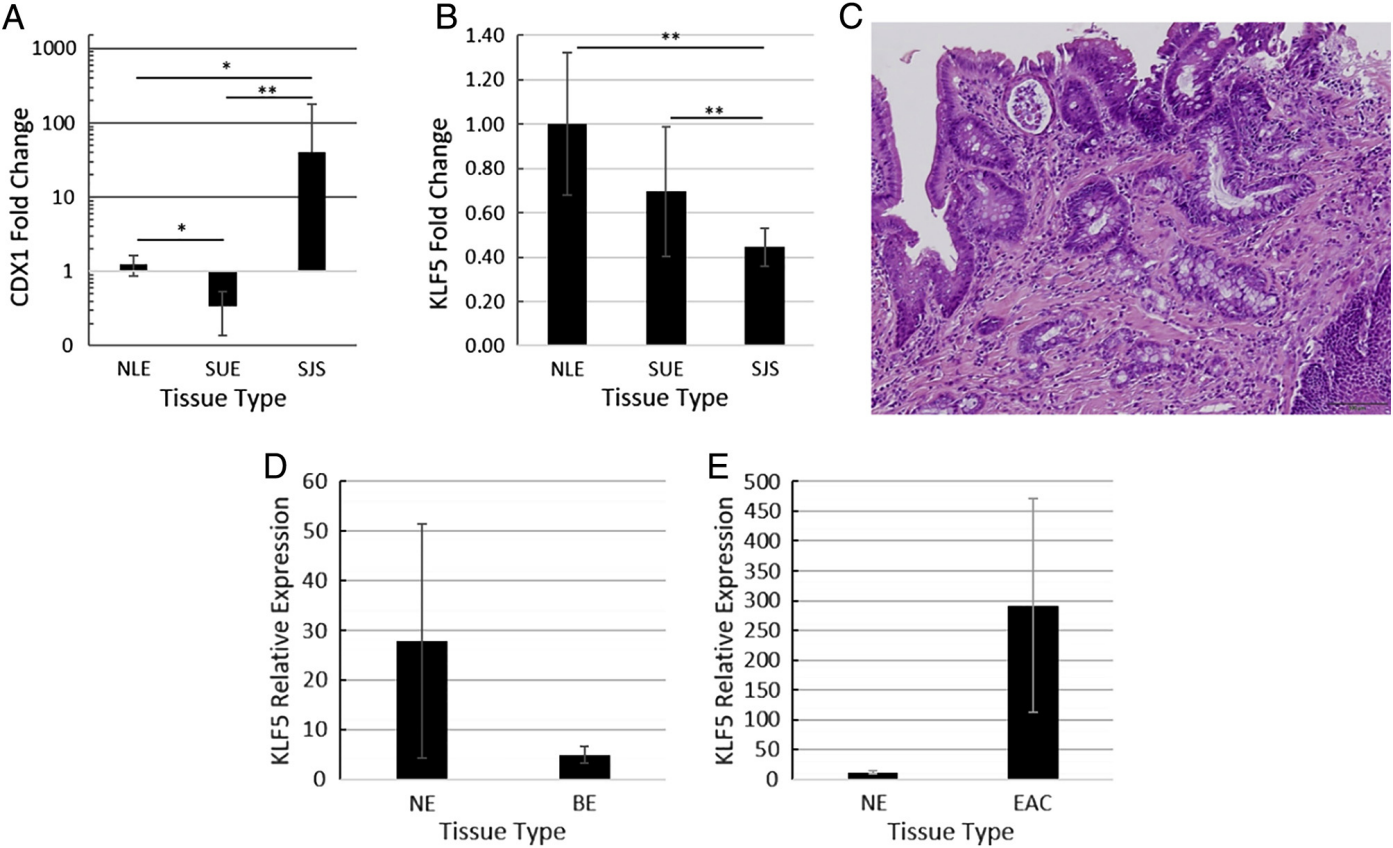
**KLF5 expression changes in rat EAC surgical model tissue.** Esophagojejunostomy was performed on rats to induce intestinal metaplasia of the lower esophagus. We performed qRT-PCR on the normal lower esophagus (NLE) of rats that underwent mock surgery, the upper esophagus (UE) of rats that underwent the surgery, and the lower esophagus near the junction site (JS) of rats that underwent the surgery. Expression levels were normalized to NLE. Error bars represent standard deviations. A) CDX1 RNA expression was significantly increased in in the lower esophagus of rats that underwent surgery. This phenotypical difference is consistent with the pathological assessment of intestinal metaplasia occurrence in these samples. No significantly changes in CDX1 expression levels was observed in the upper esophagus of rats that underwent the surgery B) KLF5 RNA expression was significantly decreased in the JS samples, consistent with the decreased KLF5 RNA expression observed in BE cell lines. CDX1 results shown in logarithmic scale for clarity; only upper error bar of SJS CDX1 results as lower error bar goes beyond a value of 0. n = 5 for NLE and SJS; n = 6 for SJS; ∗ *P* < .05; ∗∗ *P* < .001. C) Histological slide of esophageal tissue collected near esophagojejunostomy presents goblet cells and brush border, consistent with Barrett's esophagus. D) KLF5 RNA expression was decreased in BE clinical tissue when compared to matched NE clinical tissue, consistent with decreased KLF5 RNA expression observed in BE cell lines and animal tissue. E) KLF5 RNA expression was increased in EAC clinical tissue when compared to matched NE clinical tissue, consistent with the increased KLF5 RNA expression in EAC cell lines. No significant changes were observed in these experiments (*P* = .3 for NE-BE and *P* = .16 for NE-EAC). Error bars represent standard error. n = 33 NE-BE matched pairs; n = 28 NE-EAC matched pairs.

To assess the influence of KLF5 on SHH signaling and promotion of the intestinal phenotype in the esophagus, hedgehog pathway, and intestine-related gene levels were measured by qRT-PCR in esophageal samples from rats that had undergone esophagojejunostomy to induce intestinal metaplasia. We had a total of three groups: (1) Esophago-jejunal surgical junction site (SJS) tissue, (2) upper esophagus (SUE) tissue of rats that underwent surgery, and (3) normal lower esophageal (NLE) tissue of rats that did not undergo surgery. Esophago-jejunal junction site tissues (SJS) had significantly higher CDX1 expression compared to both the upper esophageal tissue of rats that underwent surgery (SUE), and the lower esophageal tissue of rats that did not undergo surgery (NLE) (Figure 4*A*); as CDX1 is expressed at high levels principally in intestinal cells and also BE tissue, these high levels of CDX1 mRNA suggested that the junction site tissues had undergone intestinal metaplasia (Figure 4*A*) [6]. Moreover, pathological assessment of junction site tissue notably exhibited goblet cells and brush border, consistent with Barrett's esophagus (Figure 4*C*).

To establish the involvement of KLF5 in esophageal adenocarcinogenesis *in vivo,* rat tissues were evaluated by qRT-PCR. These experiments revealed that SJS tissues expressed significantly lower KLF5 levels than did NLE or SUE tissues, consistent with the above BE cell line results (Figure 4*D*).

### KLF5 Expression Levels in Human Tissues

Next, we sought to determine whether KLF5 was overexpressed in human BE and EAC tissues, as it had been in BE and EAC cell lines, and murine BE model tissues. 28 matched normal esophagus (NE)-EAC tissue pairs and 33 matched NE-BE tissue pairs were assessed for KLF5 expression by qRT-PCR. Consistent with the above cell line results, KLF5 trended weakly toward downregulation in BE *vs.* matched NE (*P* = .31) and upregulation in EAC *vs.* matched NE (*P* = .18) (Figure 4*D*&E). However, these trends did not achieve statistical significance. This lack of statistical significance may have resulted from several extremely high and low KLF5 expression level outliers within the three human tissue groups.

### KLF5 Knockdown Induces Significant Changes in Cell Proliferation, Cell Migration, and Expression of EAC-Associated and SHH Pathway Genes in EAC *In Vitro*

**Figure 5:**
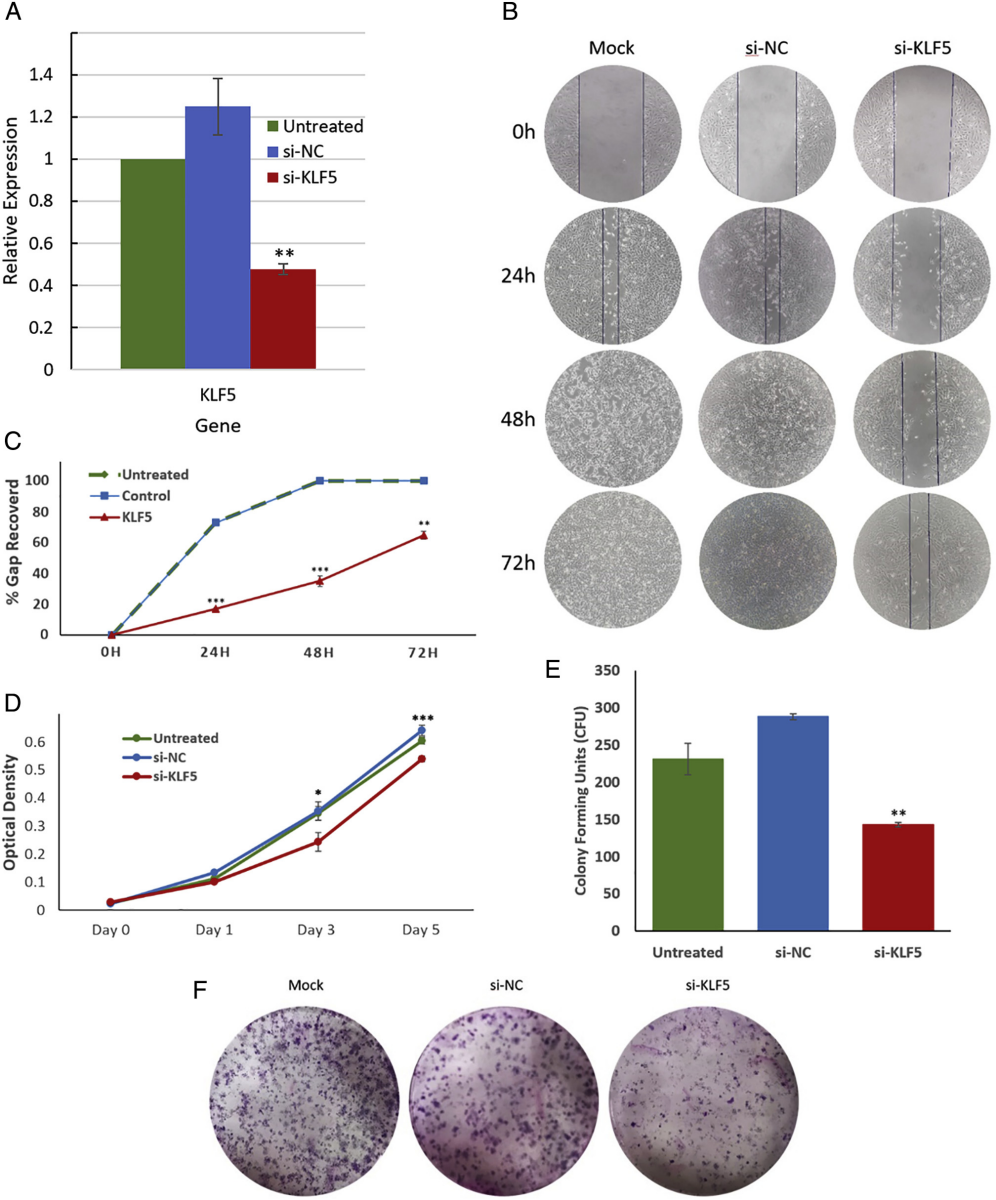
**KLF5 knockdown led functional changes *in vitro*.** A) KLF5 siRNA leads to 60% KLF5 knockdown in EAC cell line SKGT4. Expression level was normalized to vector-only SKGT4. n = 4; ∗∗*P* < .005. B) Scratch assay results of SKGT4 over the course of 72 hours. Lines are drawn to delineate the borders of the wound. Distance between the gaps was measured at the edge of the drawn lines. C) Significantly less migration was recorded in KLF5-inhibited SKGT4 cells. Error bars represent standard error. n = 4; ∗∗*P* < .005; ∗∗∗*P* < .0005. D) EAC cell line SKGT4 transfected with KLF5 siRNA was used in WST-1 assays to detect the effect of KLF5 knockdown on proliferation. Compared to control and mock-transfected cells, knocking down KLF5 significantly decreased cell proliferation 72 hours after transfection. Error bars represent standard error. n = 2; ∗ *P* < .05; ∗∗∗*P* < .0005. E and F) Significantly fewer colonies were formed by KLF5-inhibited SKGT4 cells 10 days after plating. n = 2; ∗∗*P* < .005.

Since we had shown that KLF5 was consistently upregulated in EAC cells, as well as in murine and human EAC tissues, we sought to determine whether KLF5 inhibition suppressed the neoplastic phenotype in SKGT4 EAC cells *in vitro.* KLF5 knockdown using an anti-KLF5 siRNA was successful, causing a 60% decrease in KLF5 mRNA levels *vs.* control in SKGT4 cells (Figure 5*A*).

Because KLF5 is known to be a cell cycle stimulator, we assessed the effect of KLF5 knockdown on EAC cell proliferation [28], [29], [30]. In WST-1 assays, KLF5 siRNA caused significant decreases in SKGT4 cell proliferation and cell viability at multiple time points (Figure 5*D*). Similarly, KLF5 inhibition in SKGT4 cells resulted in a significant decrease in colony formation *vs.* controls (Figure 5 E&F). Moreover, scratch assays revealed a significant decrease in SKGT4 cell migration caused by KLF5 knockdown (Fig. 5, *B* and *C*).

**Figure 6:**
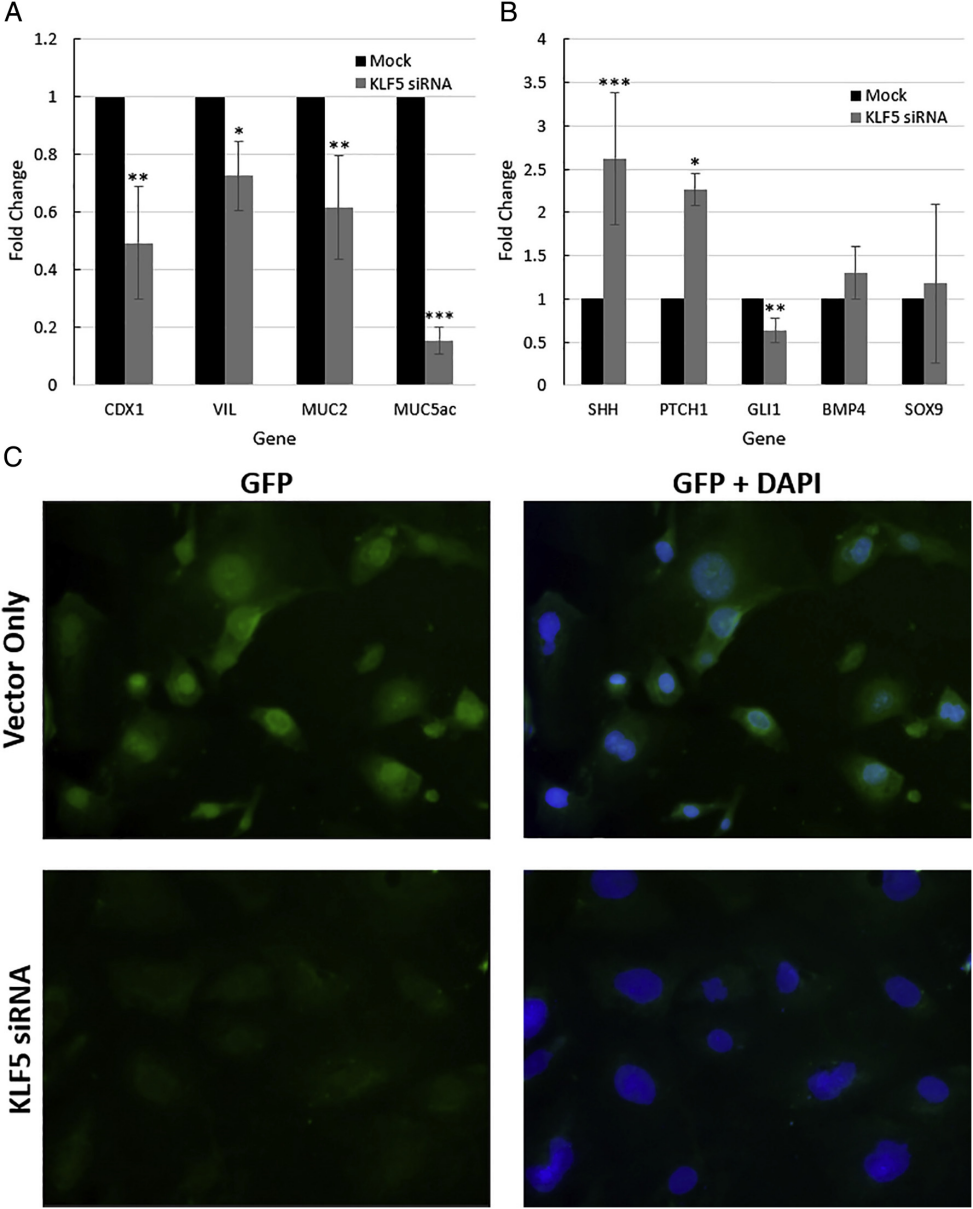
**KLF5 knockdown leads to phenotypic changes *in vitro*.** Expression levels were normalized to vector-only SKGT4. A) qRT-PCR results from KLF5 KD SKGT4 EAC cells showed significant reductions in expression levels of intestine-specific transcription factors expressed in EAC: CDX1, MUC2, MUC5ac and VIL. n = 4; ∗ *P* < .05; ∗∗ *P* < .005. ∗∗∗*P* < .0005.B) KLF5 knockdown lead to significant changes in SHH pathway genes RNA expression. GLI1, a canonical SHH pathway target gene, had significantly lower expression in response to KLF5 KD. SHH and PTCH1 RNA expressions were significantly elevated by KLF5 KD. BMP4 and SOX9 RNA expression had no significant changes due to KLF5 KD. Error bars represent standard deviations. n = 4; ∗ *P* < .05; ∗∗ *P* < .005. ∗∗∗*P* < .0005. C) GLI1 Immunocytochemistry performed on vector-only SKGT4 and KLF5-KD SKGT4. Green fluorescence is GLI1 protein; blue fluorescence is DAPI nuclear staining. Vector-only SKGT4 showed remarkable GLI1 staining in the nucleus; KLF5-KD SKGT4 lacked GLI1 nuclear staining.

We then assessed the effects of KLF5 inhibition on the intestinal phenotype in EAC. CDX1, VIL, MUC2, and MUC5ac are intestine-specific transcription factors that are also known to be upregulated in EAC [8], [10], [11]. qRT-PCR experiments showed significant downregulation of these four EAC-associated genes by KLF5 knockdown (Figure 6*A*).

Next, we measured the effect of KLF5 inhibition on SHH pathway activity. Significantly decreased expression of GLI1 and significantly increased expression of SHH and PTCH1 were observed in SHH pathway genes (Figure 6*B*). We did not observe significant changes in mRNA levels of BMP4 and SOX9, two genes which are known to be regulated by SHH in esophageal tissues and important in BE metaplasia and esophageal adenocarcinogenesis (Figure 6*B*) [20].

Finally, we used immunocytochemistry to determine whether KLF5 siRNA-induced decreases in GLI1 mRNA levels were reflected at the protein level. GLI1 protein levels were indeed lower in SKGT4 cells transfected with KLF5 siRNA *vs.* mock-transfected SKGT4 cells; moreover, GLI1 nuclear localization was clearly visible in mock-transfected SKGT4 cells, whereas almost no nuclear GLI1 protein was seen in siRNA-transfected SKGT4 cells (Figure 6*C*).

## Discussion

KLF5 is known to play a key role in intestinal maintenance, and to exert oncogenic downstream effects such as cell cycle promotion and apoptosis inhibition [28], [29], [30], [31], [32]. Our study examined KLF5's involvement in EAC, an intestinal-like cancer that develops in a squamous cell environment [3]. KLF5 dysregulation has been studied in various cancer types; however, to our knowledge, our study is the first to examine KLF5 in EAC [27], [33], [34], [35], [36], [37], [38].

KLF5 expression in BE exhibited a strikingly different pattern than in EAC, both *in vitro* and *in vivo.* The low levels of KLF5 expression we observed in BE cells and tissues may imply that BE cells and tissues resemble differentiated intestinal cells, which are known to have low KLF5 expression; conversely, higher KLF5 expression in EAC cells and tissues may reflect their similarity to less-differentiated basal intestinal epithelial cells, which express high KLF5 levels [23], [27].

Our results also suggest that KLF5 expression participates in EAC development and/or maintenance. In support of this theory, inhibition of KLF5 caused significant decreases in expression levels of intestinal- and EAC-associated genes, cell proliferation, cell migration, and colony formation. Additional experiments are required to prove that KLF5 is an esophageal oncogene, such as apoptosis assays, invasion and migration assays, and transgenic *in vivo* model studies.

A previous high-throughput screening approach identified several novel and potent small molecular inhibitors of KLF5, such as Wortmannin, AG17, and AG879, which inhibit proliferation of colon cancer cell lines [42]. It is conceivable that these inhibitors have the same effect on EAC cell lines and in murine models. Thus, further experiments to test these inhibitors are now indicated.

Moreover, studies have shown that metformin, an anti-type 2 diabetes drug, leads to KLF5 degradation [43]. As EAC is commonly co-morbid with type 2 diabetes, targeting KLF5 with metformin may represent a possible supplement to conventional EAC therapies. One potential concern is that if metformin's KLF5-degrading effect is sufficient to affect EAC cells, it may also exert adverse effects on healthy epithelial cells in the GI tract (where KLF5 normally participates in GI epithelial maintenance). Metformin has been studied extensively as a potential therapy targeting KLF5 in other cancer types, such as endometrial, prostate and breast cancer, and some metformin cancer studies have sought to target other gene pathways such as the PI3K/Akt/mTOR pathway [44], [45]. Currently, numerous clinical trials are assessing metformin as an adjuvant to conventional chemotherapy in prostate, endometrial, breast, and pancreatic cancer; collectively, metformin has been shown to have a favorable effect on tumor markers, but additional studies and time are required to assess its effect on survival rates [44], [45], [46], [47], [48].

In our experiments, knocking down KLF5 significantly altered SHH pathway gene expression levels. In particular, GLI1, a canonically specific SHH pathway target gene, was significantly downregulated by KLF5 knockdown; moreover, GLI1 protein levels and nuclear localization both diminished with KLF5 knockdown. GLI1's mRNA and protein levels were lowered despite significantly increased SHH expression caused by KLF5 knockdown. Possibly, the downstream effects of the increased SHH expression may have been negated by the significant increase in expression of PTCH1, a canonical inhibitor of the SHH pathway. These results suggest a link between KLF5 expression and SHH pathway activity; however, our results do not explain exactly where or how this inter-pathway connection occurs. Additional studies are now indicated to explore this connection.

A previous study by Wang et al. showed that the transport of SHH protein out of esophageal epithelial cells leads to BMP4 and SOX9 upregulation in esophageal stromal cells [18], [20]. SOX9 expression was found to be sufficient to drive columnar differentiation of esophageal squamous epithelium [19]. Our results are consistent with these findings, in that changes in SHH pathway activity had no significant effect on BMP4 or SOX9 levels in EAC cells. Interestingly, we found that itraconazole, an SHH pathway antagonist, induced a significant decrease in BMP4 expression in SKGT4 EAC cells [49]. The importance of this epithelial-stromal cell interaction suggests that accurately studying the SHH pathway in BE and EAC will necessitate the use of organoid and *in vivo* models, as well as clonal esophageal epithelial cell lines. Thus, while GLI1 expression and protein levels were downregulated *in vitro* by KLF5 knockdown, this finding may not be present at the tissue level, where stromal cell-derived BMP4 and SOX9 are key factors in BE and EAC development.

The above results do not permit us to definitively conclude that KLF5 affects BE and EAC development *via* SHH pathway activation. However, the significant changes observed in SHH pathway gene expression do suggest a novel connection between KLF5 and SHH signaling. This relationship may indicate a similar connection during development - wherein both genes play key roles - as well as in other cancer types, where both genes are frequently dysregulated [25], [27], [50].

In summary, this study allowed us to make several key observations. Firstly, we have shown the KLF5 is aberrantly expressed in BE and EAC *vs.* normal cells and tissues. Secondly, knocking down KLF5 led to decreases in EAC-associated genes, cell proliferation levels, and cell migration rates. Thirdly, knockdown of KLF5 led to down-regulation of SHH pathway genes and GLI1 protein levels. Taken together, our findings identify KLF5 as a potential oncogene in esophageal adenocarcinoma and suggest a novel connection between KLF5 and the SHH pathway.

The following are the supplementary data related to this article.

Supplementary table 1 Primers used for qRT-PCR Supplementary table 1

## Authors' Contributions

C.N. conceived the presented idea. C.N. and K.M. designed and carried out the experiments and analytic methods with technical advice from Y.C.. J.H. and T.M. contributed with in vivo samples. S.M. supervised the project. All authors discussed the results and contributed to the final manuscript.

## Conflict of Interest Statement

The authors declare that they have no conflict of interest.
