# Supplementary material for: Krüppel-like Factor 5 Promotes Sonic Hedgehog Signaling and Neoplasia in Barrett's Esophagus and Esophageal Adenocarcinoma
Source: Transl Oncol. 2019 Aug 8;12(11):1432–41. doi: 10.1016/j.tranon.2019.07.006 (PMC6700477; doi:10.1016/j.tranon.2019.07.006)
Supplement: Supplementary table 1 — Primers used for qRT-PCR [file mmc1.docx]

| **Primers used for qRT-PCR** | | | |
| --- | --- | --- | --- |
| **Gene** | **Species** | **Forward Primer (5'→ 3')** | **Reverse Primer (5'→ 3')** |
| GAPDH | *Homo Sapiens* | CAGCCTCAAGATCATCAGCA | TGTGGTCATGAGTCCTTCCA |
| GAPDH | *Rattus Norvegicus* | GCTGGCATTGCTCTCAATGACA | TCCACCACCCTGTTGCTGTA |
| KLF5 | *Homo Sapiens* | GGTCCAGACAAGATGTGAAATGG | TTTATGCTCTGAAATTATCGGAACTG |
| KLF5 | *Rattus Norvegicus* | AGCTCACCTGAGGACTCATA | GTGCGCAGTGCTCAGTTCT |
| CDX1 | *Homo Sapiens* | AGGACAAGTACCGCGTGGTCTA | CCTCTGAACGTATGGAGGAGGA |
| CDX1 | *Rattus Norvegicus* | AGAGCTGGCTGCTAACTTGG | CATTGGTGGGGCATAGACTC |
| VIL | *Homo Sapiens* | CCCTGGAGCAGCTAGTGAAC | ATCGAGGCAGAGCAGAGAAG |
| MUC2 | *Homo Sapiens* | CAGCACCGATTGCTGAGTTG | GCTGGTCATCTCAATGGCAG |
| MUC5ac | *Homo Sapiens* | ACCGGTGCCACATGACGGAC | ACGTGGCCGCCTCACACGTG |
| SHH | *Homo Sapiens* | GCTCGGTGAAAGCAGAGAAC | CCAGGAAAGTGAGGAAGTCG |
| PTCH1 | *Homo Sapiens* | TTCCAGCGCTTTCTACATCT | CTTTCTCGTGGACCCATTCT |
| GLI1 | *Homo Sapiens* | GTGCAAGTCAAGCCAGAACA | ATAGGGGCCTGACTGGAGAT |
| BMP4 | *Homo Sapiens* | GGCTGGAATGACTGGATTGT | TGGTTGAGTTGAGGTGGTCA |
| SOX9 | *Homo Sapiens* | AGGCAAGCAAAGGAGATGAA | TGGTGTTCTGAGAGGCACAG |
